# Near Field Differential Interference Contrast Microscopy

**DOI:** 10.1038/s41598-020-66482-z

**Published:** 2020-06-15

**Authors:** Hesam Heydarian, Payam Yazdanfar, Arezoo Zarif, Bizhan Rashidian

**Affiliations:** Department of Electrical Engineering, Sharif University of Technology, Tehran, 11365-8639 Iran

**Keywords:** Engineering, Nanoscience and technology, Optics and photonics

## Abstract

Near field scanning optical microscopy exploiting differential interference contrast enhancement is demonstrated. Beam splitting in the near field region is implemented using a dual color probe based on plasmonic color sorter idea. This provides the ability to illuminate two neighboring points on the sample simultaneously. It is shown that by modulating the two wavelengths employed in exciting such a probe, phase difference information can be retrieved through measuring the near field photoinduced force at the difference of the two modulation frequencies. This difference in frequency is engineered to correspond to the first resonant frequency of the cantilever, resulting in improved SNR, and sensitivity. The effect of both topographical and material changes in the proposed near field differential interference (NFDIC) technique are investigated for CNT and silica samples. This method is a promising technique for high contrast and high spatial resolution microscopy.

## Introduction

High resolution imaging, beyond the conventional diffraction limit has lead to the development of near-field microscopy (SNOM), in which the evanescent waves containing high spatial harmonic frequencies are collected in the near field region. Near-field microscopy, in its conventional form, delivers insufficient image contrast due to evaluating only field intensity as the image information. Inspired from far-field microscopy several contrast-enhancing techniques, including phase contrast^1,2^, polarization contrast^3^, and wavelength contrast (such as fluorescence^4^ and two photon microscopy^5^) have been proposed for improvement of near field microscopy. However differential interference contrast (DIC) microscopy has not been yet applied to SNOM.

**Figure 1:**
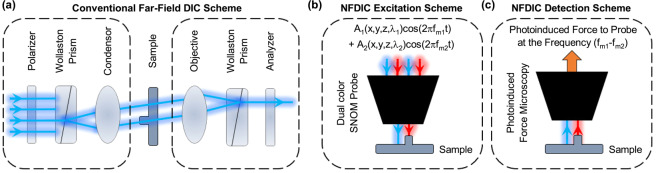
Inspiring the near-field differential interference contrast microscopy from that in conventional far-field microscopy. (**a**) Conventional far-field DIC scheme. (**b**) NFDIC excitation scheme working in a similar manner to that in far-field DIC which enables to illuminate two neighboring points on the specimen. (c) NFDIC detection scheme working in a similar manner to that in far-field DIC which enables the interference of two optical signals containing image information by photoinduced force mapping.

DIC microscopy, relying on detection of the phase change resulting from either topological or refractive index changes, is a powerful tool to provide high-contrast images using the phase information provided by the interference signal. In a conventional optical far field DIC system (Fig. 1(a)), the beam of the light source is splitted into two orthogonally polarized spatially separated beams through a Wollaston prism, to excite two adjacent points on the specimen. A difference in thickness and/or optical properties of the two distinct excited points results in different optical path lengths of the two beams, which in turn induces a phase-difference between them. Through interfering of these two beams using another Wollaston prism, the relative phase difference is converted to a measurable intensity containing phase information^6^. Therefore, DIC provides the ability in visualizing weakly scattered samples which have low contrast images in conventional intensity contrast microscopy. The same is true for sharp imaging of edges on the samples. Several challenging issues have prohibited the implementation of DIC in SNOM. These include high resolution excitation of the two neighboring points on the specimen, near-field interference of the two scattered beams from the specimen, and detection of very low phase changes resulting from nanometric changes of optical path length during the near field scanning of the specimen’s topography.

Here, we demonstrate the implementation of a near-field differential interference contrast microscopy (NFDIC) technique by the means of combining a dual color SNOM system and a photoinduced force microscope (PiFM). Diffraction-unlimited excitation of two neighboring points can be performed utilizing novel SNOM probes^7^. In spite of a great deal of work in designing innovative aperture^8–10^ and apertureless^11–13^ SNOM probes, none of them are capable of beam splitting in the near field region. Previously reported nanoantenna probes have been generally exploited to generate large near-field enhancement and optical confinement at the probe’s tip, as well as large optical throughput and detection efficiency of the probes. However, diffraction-unlimited excitation of the two neighboring points can be done through utilizing the novel SNOM probes reported in our previous works^7,14^, a new category of SNOM probes known as dual color probe. This kind of near-field plasmonic probes, relying on generating two hot spots at well-defined locations on the sample surface, is an excellent tool for splitting the beam of the light source in the near field region. The underpinning mechanism of this energy/frequency beam splitting is the hybridization of the plasmon modes due to the symmetry breaking of the nanoantenna structure on top of the probe^15^. It is worth mentioning that our proposed plasmonic dual color probe also has additional advantages features, including improvement in accuracy and speed of imaging, while doing its main job as a plasmonic nanoantenna probe providing large enhancement, and confinement of the illuminating field^7^.

The two separated hot spots created at the near-field vicinity of the probe interact with two different points of the sample, inducing a phase difference between the scattered waves. However, detecting these two scattered waves having different wavelengths in far field regions is problematic. The phase difference resulting from the small optical path length difference would be negligible with respect to the relatively large induced phase change at a large distance. Hence, we have to combine the two signals in the near field region. In addition, ordinary interference techniques cannot extract this phase difference, unless the two interference signals have the same wavelength and polarization. To do so, we propose exploiting photoinduced forces generated in the near field region of each signal, and interfering them. Extracting near field optical forces are the basis of the photoinduced force microscopy^16–18^, in which optically-induced localized polarizability of the specimen is measured by mechanical detection of the force gradient generated due to the interaction between a sharp polarizable tip and the specimen. Since in the PiFM the detection is carried out in the near-field region, it can be employed in our proposed NFDIC system to interfere non monochromatic scattered fields from the sample (Fig. 1(c)). By using the idea behind previously reported near field phase contrast microscopy^2^, in which optical phase changes are extracted by modulating incident signal and employing standard interferometry techniques, phase changes in photoinduced forces in the near field region can be obtained. However, in the NFDIC system, it is necessary to have two distinguishable incident signals. Here we use two signals with different wavelengths which are modulated with two different frequencies for extracting the interference signal as explained in more details below.

## Theory: Proof of Concept

In order to show the possibility of interfering two optical signals with different colors ${\lambda }_{1}$ and ${\lambda }_{2}$ in the near field regions by photoinduced forces, we calculate the total force applied to the probe. In this way, we modulate the two excitation signals, coupled into the probe, with a specific frequency, and extract the interference data from the induced force signal. As shown in Fig. 1(b), two external lasers, modulated with frequencies ${f}_{m1}$ and ${f}_{m2}$, are coupled to the dual color SNOM probe, which excite two laterally separated points on the sample surface. Exploiting the high quality factor (Q) of the cantilever beam, the values of ${f}_{m1}$ and ${f}_{m2}$ are chosen so that the difference between these modulation frequencies matches with the fundamental mechanical resonance frequency of the cantilever (${f}_{m1}-{f}_{m2}={f}_{0}$). The detection process is also carried out at this resonant frequency, resulting in large improvements of sensitivity and signal to noise ratio (SNR). We assume, without losing the generality, the time harmonic electric and magnetic field of the two illumination signals are in the form of:

1 $$\begin{array}{ll} & {{\bf{E}}}_{\alpha }({\bf{r}},t)=Re\{{{\bf{E}}}_{\mathrm{0,}\alpha }({\bf{r}}){e}^{-i2\pi ({f}_{\alpha }+{f}_{{m}_{\alpha }})t}\}\\  & {{\bf{H}}}_{\alpha }({\bf{r}},t)=Re\{{{\bf{H}}}_{\mathrm{0,}\alpha }({\bf{r}}){e}^{-i2\pi ({f}_{\alpha }+{f}_{{m}_{\alpha }})t}\}\end{array}$$

where ${{\bf{E}}}_{\mathrm{0,}\alpha }({\bf{r}})$ is the spatial distribution of the ${\alpha }^{th}$ $(\alpha =\mathrm{1,2)}$ excitation at the wavelength of ${\lambda }_{\alpha }$ (frequency ${f}_{\alpha }$) which is modulated with the frequency of ${f}_{{m}_{\alpha }}$. Next, we employ a general approach of calculating photoinduced force by considering both the surface integral of the Maxwell stress tensor and the effect of electromagnetic field momentum to get a relatively accurate estimate of the optical force (**F**) experienced by the tip^19^:

2 $${\bf{F}}={\int }_{S}\overleftrightarrow{{\bf{T}}}({\bf{r}},t).{\bf{n}}({\bf{r}})ds-\frac{1}{{c}^{2}}\frac{d}{dt}{\int }_{V}{\bf{E}}\times {\bf{H}}dv$$

where *S* is an arbitrary surface enclosing the tip, $n$ is the unit vector perpendicular to the surface, $V$ is the volume surrounded by $S$, and $\overleftrightarrow{{\bf{T}}}({\bf{r}},t)$ stands for the Maxwell stress tensor, which is calculated from the total electric $({\bf{E}})$ and magnetic $({\bf{H}})$ fields as:

3 $$\overleftrightarrow{{\bf{T}}}={\varepsilon }_{0}\varepsilon {\bf{EE}}-{\mu }_{0}\mu {\bf{HH}}-\frac{1}{2}({\varepsilon }_{0}\varepsilon {E}^{2}+{\mu }_{0}\mu {H}^{2})\overleftrightarrow{{\bf{I}}}$$

where ${\varepsilon }_{0}\varepsilon$ and ${\mu }_{0}\mu$ are electric permittivity and magnetic permeability of the surface S and $\overleftrightarrow{{\bf{I}}}$ is the unit tensor. Therefore, Maxwell stress tensor can be calculated by inserting ${E}_{\beta \alpha }({\bf{r}},t)=Re\{{E}_{\mathrm{0,}\beta \alpha }({\bf{r}})\exp (\,-\,i\mathrm{(2}\pi ({f}_{\alpha }+{f}_{{m}_{\alpha }})t+{\phi }_{\beta \alpha }))\}$, which stands for time modulated total electric field in *β* direction (*β* = *x*, *y*, *z*) and generated by the *α*^*th*^ hot spot, in Eq. (3). Knowing that cantilever responds only to z-direction forces, the photoinduced force in z-direction resulting from the first hot spot (${F}_{z1}$) can be described as:

4 $$\begin{array}{rcl}{F}_{z1} & = & \frac{\varepsilon {\varepsilon }_{0}}{2}{\int }_{yz}{E}_{\mathrm{0,}x1}{E}_{\mathrm{0,}z1}\{\,\cos \,\mathrm{(4}\pi ({f}_{1}+{f}_{{m}_{1}})t+{\phi }_{z1}+{\phi }_{x1})+\,\cos ({\phi }_{z1}-{\phi }_{x1})\}ds\\  &  & +\frac{\varepsilon {\varepsilon }_{0}}{2}{\int }_{xz}{E}_{\mathrm{0,}y1}{E}_{\mathrm{0,}z1}\{\,\cos \,\mathrm{(4}\pi ({f}_{1}+{f}_{{m}_{1}})t+{\phi }_{z1}+{\phi }_{y1})+\,\cos ({\phi }_{z1}-{\phi }_{y1})\}ds\\  &  & +\frac{\varepsilon {\varepsilon }_{0}}{4}{\int }_{xy}\{{E}_{\mathrm{0,}z1}^{2}\,\cos \,\mathrm{(4}\pi ({f}_{1}+{f}_{{m}_{1}})t+2{\phi }_{z1})-{E}_{\mathrm{0,}x1}^{2}\,\cos \,\mathrm{(4}\pi ({f}_{1}+{f}_{{m}_{1}})t+2{\varphi }_{x1})\\  &  & -{E}_{\mathrm{0,}y1}^{2}\,\cos \,\mathrm{(4}\pi ({f}_{1}+{f}_{{m}_{1}})t+2{\phi }_{y1})+({E}_{\mathrm{0,}z1}^{2}-{E}_{\mathrm{0,}x1}^{2}-{E}_{\mathrm{0,}y1}^{2})\}ds-\frac{1}{{c}^{2}}\frac{d}{dt}{\int }_{V}{({{\bf{E}}}_{1}\times {{\bf{H}}}_{1})}_{z}dv\end{array}$$

where ${E}_{\mathrm{0,}\beta 1}$ and ${\phi }_{\beta 1}$ are the electric field spatial distribution and phase difference generated due to the interaction of the first source with sample in $\beta$ direction, respectively. Here, as we deal with non-magnetic environments, we neglect the effect of magnetic field on the Maxwell stress tensor. Similar to Eq. (4), an equation for z-direction photoinduced force $({F}_{z2})$ can be derived when the second hot spot illuminates the sample. To extract the interference signal, total photoinduced force $({F}_{zt})$ applied to the probe resulting from simultaneous excitation of the sample with two hot spots should be considered. By inserting ${E}_{x}={E}_{x1}+{E}_{x2}$, ${E}_{y}={E}_{y1}+{E}_{y2}$ and ${E}_{z}={E}_{z1}+{E}_{z2}$ in Eq. (3), ${F}_{zt}$ can be described in terms of ${F}_{z1}$, ${F}_{z2}$ and other terms representing interaction between two optical signals:

5 $$\begin{array}{rcl}{F}_{zt} & = & {F}_{z1}+{F}_{z2}+\varepsilon {\varepsilon }_{0}{\int }_{yz}\{{E}_{x1}{E}_{z2}+{E}_{x2}{E}_{z1}\}ds+\varepsilon {\varepsilon }_{0}{\int }_{xz}\{{E}_{y1}{E}_{z2}+{E}_{y2}{E}_{z1}\}ds\\  &  & +\varepsilon {\varepsilon }_{0}{\int }_{xy}\{{E}_{z1}{E}_{z2}-{E}_{x1}{E}_{x2}-{E}_{y1}{E}_{y2}\}ds-\frac{1}{{c}^{2}}\frac{d}{dt}{\int }_{V}{({{\bf{E}}}_{1}\times {{\bf{H}}}_{2}+{{\bf{E}}}_{2}\times {{\bf{H}}}_{1})}_{z}dv\end{array}$$

Considering that the modulation frequencies are chosen in a way that difference between them ($\Delta {f}_{m}={f}_{{m}_{1}}-{f}_{{m}_{2}}$) matches with the fundamental mechanical resonance of the cantilever, we keep those terms of Eq. (5) in which $\Delta {f}_{m}$ appears. It is worth noting that for these terms, having low optical frequency of ${f}_{1}-{f}_{2}$, the volume integral of electromagnetic field momentum can be ignored. This assumption would be stronger given that the photoinduced force in our proposed aperture based illumination system is mainly resulted from extremely high electric field divergence. The interference force (${F}_{{z}_{NFDIC}}$) can thus be:

6 $$\begin{array}{rcl}{F}_{{z}_{NFDIC}} & = & \frac{\varepsilon {\varepsilon }_{0}}{4}{e}^{(-i2\pi (\Delta f+\Delta {f}_{m})t)}\{{\int }_{xy}\{{E}_{\mathrm{0,}z1}{E}_{\mathrm{0,}z2}{e}^{-i({\phi }_{z1}-{\phi }_{z2})}-{E}_{\mathrm{0,}x1}{E}_{\mathrm{0,}x2}{e}^{-i({\phi }_{x1}-{\phi }_{x2})}-{E}_{\mathrm{0,}y1}{E}_{\mathrm{0,}y2}{e}^{-i({\phi }_{y1}-{\phi }_{y2})}\}ds\\  &  & +{\int }_{yz}\{{E}_{\mathrm{0,}x1}{E}_{\mathrm{0,}z2}{e}^{-i({\phi }_{x1}-{\phi }_{z2})}+{E}_{\mathrm{0,}x2}{E}_{\mathrm{0,}z1}{e}^{-i({\phi }_{z1}-{\phi }_{x2})}\}ds+{\int }_{xz}\{{E}_{\mathrm{0,}y1}{E}_{\mathrm{0,}z2}{e}^{-i({\phi }_{y1}-{\phi }_{z2})}+{E}_{\mathrm{0,}y2}{E}_{\mathrm{0,}z1}{e}^{-i({\phi }_{z1}-{\phi }_{y2})}\}ds\}+C\mathrm{}.C\mathrm{}.\end{array}$$

The inertia of mechanical vibration and the slow response of the cantilever compared to the much higher frequency optical signal leads to detection of the envelope of these optical frequency variations:

7 $$\begin{array}{rcl}{F}_{{z}_{NFDIC}} & = & \frac{\sqrt{2}\varepsilon {\varepsilon }_{0}}{2}\{{\int }_{yz}\left\{{E}_{\mathrm{0,}x1}{E}_{\mathrm{0,}z2}\,\cos \left(2\pi \Delta {f}_{m}t+{\phi }_{x1}-{\phi }_{z2}+\frac{\pi }{4}\right)+{E}_{\mathrm{0,}x2}{E}_{\mathrm{0,}z1}\,\cos \left(2\pi \Delta {f}_{m}t+{\phi }_{x2}-{\phi }_{z1}+\frac{\pi }{4}\right)\right\}ds\\  &  & +{\int }_{xz}\left\{{E}_{\mathrm{0,}y1}{E}_{\mathrm{0,}z2}\,\cos \left(2\pi \Delta {f}_{m}t+{\phi }_{y1}-{\phi }_{z2}+\frac{\pi }{4}\right)+{E}_{\mathrm{0,}y2}{E}_{\mathrm{0,}z1}\,\cos \left(2\pi \Delta {f}_{m}t+{\phi }_{y2}-{\phi }_{z1}+\frac{\pi }{4}\right)\right\}ds\\  &  & +{\int }_{xy}\left\{{E}_{\mathrm{0,}z1}{E}_{\mathrm{0,}z2}\,\cos \left(2\pi \Delta {f}_{m}t+\Delta {\phi }_{z}+\frac{\pi }{4}\right)-{E}_{\mathrm{0,}x1}{E}_{\mathrm{0,}x2}\,\cos \left(2\pi \Delta {f}_{m}t+\Delta {\phi }_{x}+\frac{\pi }{4}\right)-{E}_{\mathrm{0,}y1}{E}_{\mathrm{0,}y2}\,\cos \left(2\pi \Delta {f}_{m}t+\Delta {\phi }_{y}+\frac{\pi }{4}\right)\right\}ds\}\end{array}$$

where $\Delta {\phi }_{\beta }$ ($\beta =x,y,z$) is equal to ${\phi }_{\beta 1}-{\phi }_{\beta 2}$. These equations clearly demonstrate that by converting two optical signals to photoinduced forces, the interference signal in the near field regions can be retrieved at the difference modulation frequency $(\Delta {f}_{m})$. In the following, we try to derive a very simplified relation between the phase extracted by interfering forces, and optical phase. First, we neglect the electric fields in x and y direction compared to the z component, due to the larger polarizability of the tip in the z-direction:

8 $$\begin{array}{l}{F}_{{z}_{NFDIC}}=\frac{\sqrt{2}\varepsilon {\varepsilon }_{0}}{2}{\int }_{xy}{E}_{\mathrm{0,}z1}{E}_{\mathrm{0,}z2}cos\left(2\pi ({f}_{m1}-{f}_{m2})t+({\varphi }_{z1}-{\varphi }_{z2}+\frac{\pi }{4})\right)ds\end{array}$$

For simplicity, it is assumed that the two sources are localized in the region with the area of $\Delta x\Delta y$ ($\Delta x,\Delta y\to 0$), therefore, the total optical force is primarily induced by the local electric fields in the locations of the first $({r}_{1})$ and second $({r}_{2})$ point sources. Therefore, by demodulating total z-direction optical force at the first mechanical resonance frequency of the cantilever ${f}_{0}={f}_{m1}-{f}_{m2}$, we finally find the interference force $({F}_{z,NFDIC})$ in terms of the relative phase difference generated by the sample $(\Delta \varphi )$ as:

9 $$\begin{array}{l}{F}_{z,NFDIC}=\frac{\sqrt{2}\varepsilon {\varepsilon }_{0}}{2}\Delta x\Delta y{F}_{ac}\,\cos \,\mathrm{(2}\pi ({f}_{m1}-{f}_{m2})t+\Delta \phi )\end{array}$$

here ${F}_{ac}$ and $\Delta \phi$ for the two point sources are given by:

10 $$\begin{array}{ll} & {F}_{ac}^{2}={E}_{\mathrm{0,}z1}^{2}({r}_{1}){E}_{\mathrm{0,}z2}^{2}({r}_{1})+{E}_{\mathrm{0,}z1}^{2}({r}_{2}){E}_{\mathrm{0,}z2}^{2}({r}_{2})+2{E}_{\mathrm{0,}z1}({r}_{1}){E}_{\mathrm{0,}z2}({r}_{1}){E}_{\mathrm{0,}z1}({r}_{2}){E}_{\mathrm{0,}z2}({r}_{2})cos(\Delta {\phi }_{z}({r}_{1})-\Delta {\phi }_{z}({r}_{2}))\\  & \Delta \phi =arctan\left(\frac{{E}_{\mathrm{0,}z1}({r}_{1}){E}_{\mathrm{0,}z2}({r}_{1})sin(\Delta {\phi }_{z}({r}_{1})+\frac{\pi }{4})+{E}_{\mathrm{0,}z1}({r}_{2}){E}_{\mathrm{0,}z2}({r}_{2})sin(\Delta {\phi }_{z}({r}_{2})+\frac{\pi }{4})}{{E}_{\mathrm{0,}z1}({r}_{1}){E}_{\mathrm{0,}z2}({r}_{1})cos(\Delta {\phi }_{z}({r}_{1})+\frac{\pi }{4})+{E}_{\mathrm{0,}z1}({r}_{2}){E}_{\mathrm{0,}z2}({r}_{2})cos(\Delta {\phi }_{z}({r}_{2})+\frac{\pi }{4})}\right)\end{array}$$

For a case of having two similar point sources which scan the same sample, we can consider ${E}_{\mathrm{0,}z1}({r}_{1}){E}_{\mathrm{0,}z2}({r}_{1})={E}_{\mathrm{0,}z1}({r}_{2}){E}_{\mathrm{0,}z2}({r}_{2})$, and $({\phi }_{z1}({r}_{1})-{\phi }_{z2}({r}_{1}))=-\,({\phi }_{z1}({r}_{2})-{\phi }_{z2}({r}_{2}))$, so that the phase difference $\Delta \phi$ extracted by NFDIC system will be equal to $\pi \mathrm{/4}$. This fixed value of phase can be obviously taken into account in simple calibration of the output data. Moreover, considering Eq. 10 in a simple case of having single wavelength illumination (ignoring the effect of the second hot spot), we can actually correspond the real optical phase induced by the first illuminating wavelength to that for photoinduced force.

## Results

In this section, we demonstrate the feasibility of implementing NFDIC, and numerically characterize different specimens through this method. After describing the dual color probe exploited as the beam splitter, first we show that the proposed method results in a calculated phase difference proportional to a step height on the sample surface. The linear behavior observed clearly resembles what we would expect from a conventional DIC. It will be shown that the method has a dynamic range limited by fundamental SNOM limitations. In the next step, the capability of this method for imaging the change in material properties is demonstrated by applying it in imaging a biological sample without topographical variations, a silica nano rod, and a CNT sample. Finally, performance of the NFDIC system in comparison with the conventional SNOM systems will be evaluated.

### Illumination system in the NFDIC microscopy

**Figure 2:**
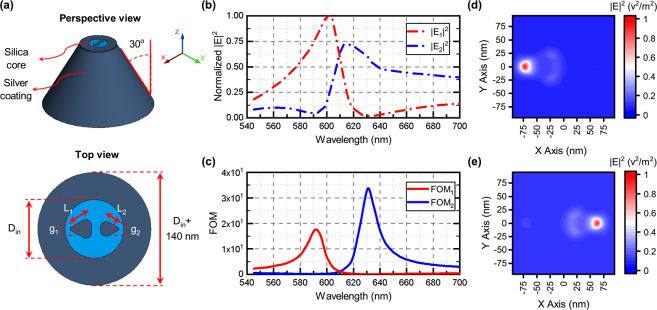
Investigating of dual color probe behavior as a near-field beam splitter. (**a**) Three-dimensional perspective view and top view of the dual color SNOM probe. (**b**) Spectrum of maximum local electric field intensity in the 5 nm distance from the left ($|{E}_{max1}{|}^{2}$ at region ${g}_{1}$) and right ($|{E}_{max2}{|}^{2}$ at region ${g}_{2}$) hand side gaps, (**c**) and corresponding local electric field intensity ratio $FO{M}_{1}=|{E}_{max1}/{E}_{max2}{|}^{2}$ and $FO{M}_{2}=|{E}_{max2}/{E}_{max1}{|}^{2}$. Normalized electric field intensity in the 5 nm distance from the probe’s tip for excitation wavelengths of (**d**) 632.8 nm and (**e**) 594.1 nm.

Figure 2(a) presents the schematics of the proposed dual-color plasmonic SNOM probe (which acts as a plasmonic near-field splitter), composed of two asymmetric equilateral silver triangles embedded in a silica ($n=1.5$) disk superimposed on top of a truncated silica fiber cone, with a cone angle of 30 degrees. The silica fiber cone as well as the silica disk on top of it are both surrounded with 70 nm-thick Ag coating. The diameter of the silica disk (probe’s opening, ${D}_{in}$) is 150 nm, and its thickness, being the same as that of the triangles, is 10 nm. The side lengths of the triangles, ${L}_{1}$ and *L*_2_, are assumed to be 70 nm and 60 nm, respectively. The vertices of triangles are assumed to be rounded with the diameter of 10 nm, in order to better model the practical structure. The gaps between each triangle and the edge of the probe’s opening is 10 nm. The excitation waves are assumed to be the fundamental mode (HE11) of the probe with two specific wavelengths (${\lambda }_{1}$ and ${\lambda }_{2}$). As a result of the coupling of two color light beams into the probe, two spatially separated hot spots can be generated in the gap regions (${g}_{1}$ and ${g}_{2}$ regions). Thus, the proposed dual color SNOM probe acts as a plasmonic near-field splitter.

The geometrical parameters of the dual color probe are designed so that the two hot spots are generated at the wavelengths of ${\lambda }_{1}=632.8$ nm and ${\lambda }_{2}=594.1$ nm, the wavelengths of a He-Ne laser. To validate that the two hot spots are spatially separated at the desired wavelengths, we show that at the excitation wavelengths of each hot spots, the effect of the other one is negligible. For this purpose, we define the figure of merit (FOM) as the ratio of the resulting electric field intensities at the two gap regions ($FO{M}_{\mathrm{1(2)}}=|{E}_{max\mathrm{1(2)}}/{E}_{max\mathrm{2(1)}}{|}^{2}$), where the two hot spots are localized for the two wavelengths. Due to the excitation of gap mode plasmons in ${g}_{1}$ and ${g}_{2}$ regions, comparing amplitude of the electric fields at these regions is desired. The large values (typically larger than 10) of the $FO{M}_{\mathrm{1(2)}}$ guarantees the existence of one hot spot in the gap region ${g}_{\mathrm{1(2)}}$ at the wavelength ${\lambda }_{\mathrm{1(2)}}$.

The maximum electric field intensity spectra in the left (region ${g}_{1}$) and right (region ${g}_{2}$) gap regions on top of the probe, and the two local electric field intensity ratio, correspond to the two FOM parameters, are plotted in Fig. 2(b,c). As can be seen in Fig. 2(c), the maximum values of the $FO{M}_{1}$ and $FO{M}_{2}$ parameters are tuned to be at the He-Ne laser wavelengths, ${\lambda }_{1}=632.8$ nm, and ${\lambda }_{2}=594.1$ nm, respectively. Two-dimensional maps of the local electric field intensities in the 5 nm distance from the probe’s tip are displayed in Fig. 2(d,e), for the desired wavelengths. The occurrence of two 15 nm hot spots (120 nm apart) in the gap regions is clear. These results illustrate the achievement of the role of a beam splitter in near field, needed for implementing NFDIC. In addition to high spatial resolution of about 15 nm and high field enhancement, other advantages of this probe are improvement in accuracy and doubling the speed of imaging^7^. The polarization sensitive behavior of the asymmetric apertureless probe has been addressed previously^20,21^, which is also the case in our proposed probe, due to the utilizing asymmetric plasmonic color sorter on top of the probe. However, our probe is tolerant enough in this regard^7^. Considering the fact that the NFDIC method presented here is not limited to the probe we have used, further improvement in polarization behavior can be achieved using two color SNOM probe based on other types of plasmonic nano structures having lower polarization sensitivity.

### Collection system in the NFDIC microscopy

**Figure 3:**
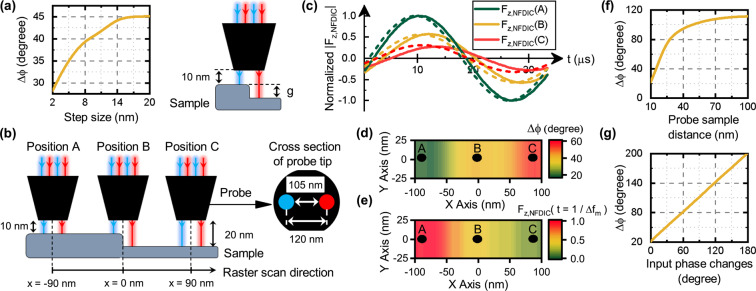
Topographical changes effect on the NFDIC signal. (**a**) Phase changes versus step size in characterizing a silica step. (**b**) Three different scenario in characterizing silica step by dual color probe, including positions A and C (points A and C in (**d**,**e**)) where both nano sources illuminate upper and lower surface of the step, respectively, and position B where one of the nano sources illuminate upper surface while the other one illuminates the lower surface of the step (point B in (**d**,**e**)). (**c**) Normalized time varying NFDIC forces corresponding to the three different positions shown in (**b**), extracted by numerical method (solid lines) and Eq. 7 (dash lines). (**d**) Phase changes, and **(e**) NFDIC (${F}_{z,NFDIC}$) mapping of the silica step with step size of 10 nm. (**f**) Phase vs probe sample distance in characterizing silica flat surface. (**g**) NFDIC phase changes in position A by sweeping the optical phase of one of the light beams.

Thus far, we have shown that the excitation of two closely-spaced points on the sample surface beyond the diffraction limit can be achieved. In the next step, to demonstrate the result of mitigating the second challenge, i.e. extracting the interference data, we start characterizing a sharp silica step (Fig. 3(a)) with a step size assumed to be name **g**. In these calculations near-field hot spots are modulated with the frequencies of 40.03 MHz and 40 MHz, enabling the detection process to occur at the frequency of 30 kHz (${f}_{0}={f}_{m1}-{f}_{m2}=30$ kHz), corresponding to the fundamental mechanical resonance frequency of the cantilever. By changing the value of **g** from 2 to 20 nm (as shown in Fig. 3(a) by keeping the distance of left surface at constant value of 10 nm), the calculated phase difference plotted in Fig. 3(a) clearly demonstrates the validity of our proposed method. In this figure deviation from the linear behavior at large spacings, occurs due to change in the probe-sample interaction, corresponding to the same fundamental dynamic range limitation in ordinary SNOM.

To study and describe the scanning procedure, we calculate the phase information of the silica sample with the step size of 10 nm for a scan area of 200 nm $\times$ 50 nm. Details of the scanning scenario are depicted in Fig. 3(b). At position A (where both hot spots shown in blue and red are spaced 10 nm above the sample), a phase shift in the detected time varying signal shown in green in Fig. 3(c) is observed which is equal to 21 degrees. This is also depicted in phase map of Fig. 3(d). As we go ahead to position of $x=-\,60$ nm, the red beam passes the step and a phase change occurs. These phase change along with NFDIC force map in positions like B (in which red beam illuminates the upper surface while the blue beam illuminates the lower surface of the step) are shown in Fig. 3(d),(e), respectively. The corresponding normalized time varying detected signal at frequency of 30 KHz extracted by numerical method and Eq. 7 are also depicted in Fig. 3(c). The next phase change will occur when the blue beam also passes the step at positions like C, inducing the phase change of 59 degrees marked in Fig. 3(d). So that for each step, we detect two phase changes (both being equal for ideal point sources) in each scan line. Knowing that the spacing between these two phase changes is always constant and is equal to about 120 nm (center-to-center distance between two hot spots), the additional jump in the extracted phase map can be readily removed by a simple data processing. It is worth to mention that the smooth local changes (about 15 nm) in the phase jumps shown in Fig. 3(d) around $x=-\,60$ nm and $x=60$ nm are due to the fact that the two hot spots on top of the probe have non zero dimensions (about 15 nm $\times$ 15 nm).

Another issue worthed to be mentioned is that by increasing the probe sample distance, in situations such as positions A and C in Fig. 3(b) in which the two hot spots are located in the same distance from the sample, one would expect having no change of the detected phase for ideal point sources. Figure 3(f) depicts that contrary to this expectation, we actually measure an increase in the detected phase changes by increasing the probe sample distance. The reason for this behavior is having two non ideal nano sources, leading to a slight difference in variation of the electric field spatial distribution between them, as the probe sample interaction is reduced by increasing the spacing. In fact we can take advantage of this behavior for the two modes usually employed in SNOM. In constant height mode, that the probe sample spacing is fixed through the closed feedback loop, one can get high contrast edge detection, as discussed previously. In open loop mode, as the probe sample spacing changes, one can store the phase versus distance data of Fig. 3(f) and detect not only topographic edges, but also difference between various probe sample distances, using phase information (in contrast to the intensity information in ordinary SNOM). Another interesting fact is depicted in Fig. 3(g), demonstrating the linear change in the detected phase difference by sweeping the optical phase of one of the input light beams, which can be utilized for system calibration and removing fixed phase change theoretically expected for ideal point sources.

**Figure 4:**
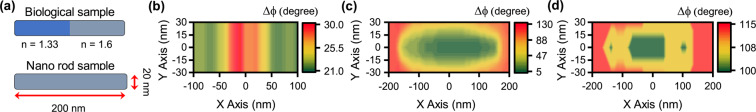
Material changes effect on the NFDIC signal. (**a**) Scheme of biological sample, silica nanorod and CNT sample under the test. (**b**) Phase changes map of the biological sample without topographical variations, in xy plane. (**c**) Phase changes map of the silica sample in xy plane. (**d**) Phase changes map of the CNT sample in xy plane.

To evaluate the effect of change in the sample material properties, we first consider a biological sample ($n=1.6$)^22^ shown in Fig. 4(a), which is in contact with water ($n=1.33$) without topographical variations. NFDIC phase changes in this sample is shown in Fig. 4(b) in which two jumps occur when each hot spot passes the boundary between water and biological sample. We also consider more complex structures including a silica rod and a carbon nanotube (CNT) (Fig. 4(a)) to evaluate the material changes effect. The length and the diameter of the silica rod are 200 nm and 20 nm, respectively. The length and outer diameter of the CNT structure is assumed to be the same as that of the silica rod. We have characterized the silica rod and the CNT structure with the NFDIC method, for a scan area 400 nm $\times$ 60 nm. Here, in order to evaluate only the effect of material changes, we characterize these two geometrically similar samples without considering substrate, at a constant probe-sample spacing of 10 nm. Figure 4(c,d) represent the phase changes for the silica rod and the CNT structure. Two major phase jumps, corresponding to the two geometrical steps in the structures are evident in these figures. Despite of the 200 nm length of the samples, the measured length in NFDIC maps (Fig. 4(c,d)) is about 120 nm larger due to the different scanning scenario explained previously. The intense jumps at the edges of the phase information, about 115 degrees jump for the silica rod (Fig. 4(b)) and 13 degrees jump for the CNT sample (Fig. 4(c)), demonstrate the high sensitivity mapping of the proposed NFDIC method. By comparing the results of these two structures in Fig. 4, it can be also seen that change in the optical properties of the sample results in a different change in the extracted phase, confirming the capability of measuring the difference in the optical properties of the same geometrical structures. Obviously the calibration data for each material can be extracted from the phase versus distance curve discussed previously.

### Performance of the NFDIC system

**Figure 5:**
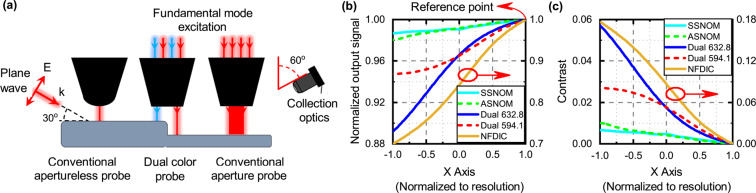
Performance improvement of the near field microscopy using NFDIC system. (**a**) Scheme of imaging silica step sample using conventional aperture SNOM probe, apertureless SNOM probe, and dual color SNOM probe. (**b**) Mapping far field optical intensity extratced by conventional aperture SNOM (ASNOM), apertureless SNOM (SSNOM), dual color probe operating at the wavelength of 632.8 nm, and dual color probe operating at the wavelength of 594.1 nm, in comparison with the phase changes extracted by NFDIC system, in a range of their corresponding resolution. (**c**) Comparison of contrast (output signal in each point compared to that of the reference point shown in (**b**)) for different imaging systems of (**a**).

To evaluate the performance of the NFDIC system, a comparison with conventional aperture and apertureless SNOM systems in characterizing a silica step is provided in Fig. 5. Here, we consider that an aperture SNOM probe with aperture size of 150 nm is excited with fundamental mode of the probe at wavelength of 632.8 nm and far field scattered signal is collected at the angle of 60 degrees with respect to the probe axis (Fig. 5(a)). To model apertureless SNOM system, we consider a conic silver probe with the tip size of 15 nm which is excited with a plane wave illuminated at the angle of 60 degrees with respect to the probe axis and extract far field scattered signal at the angle of 60 degrees with respect to probe axis in opposite direction (Fig. 5(a)). Moreover, three different approaches of imaging with the dual color probe are also considered. The first approach is imaging with the proposed NFDIC method as discussed above in detail. In the second approach, near field optical image can be achieved at wavelength of 632.8 nm by illuminating the sample surface with the first hot spot, and extracting scattered field with a color sensitive sensor operating in the wavelength ranges around 632.8 nm (in the similar configuration to that of conventional aperture SNOM system). This system works under the same conditions (same optics and instruments) as the conventional aperture SNOM system, but with better resolution, due to field confinement achieved by utilizing plasmonic nanostructure on top of the tip of probe. Its performance is comparable to the previously reported state of the art aperture SNOM probes^8–10^. The third approach is extracting near field optical image at wavelength of 594.1 nm by illuminating the sample surface with the second hot spot, and extracting scattered field in far field with another color sensitive sensor operating in the wavelength ranges around 594.1 nm (Fig. 5(a)). This image is independent of the image extracted using the first hot spot, and provides a redundant imaging data, with a little shift in time frame compared to that obtained with the first hot spot (due to the fixed 120 nm spacing between two hot spots). These two data sets of sample information which are obtained in a single mechanical scanning procedure, makes it possible to decrease the instrumental artifacts such as thermal drift and creep of the positioning system, as discussed in our previous report^7^. In order to better compare the different imaging schemes, performance of the above mentioned systems in imaging a silica step is evaluated in a range of their corresponding resolution with respect to the step (by finding the variations of the outputs of neighboring points separated by 150 nm for aperture SNOM, and 15 nm for apertureless SNOM, dual color aperture SNOM and NFDIC which corresponds to the resolution of these systems). Figure 5(b),(c) represent the signal detected by the output sensor and contrast obtained with different imaging systems discussed above, respectively. Here, contrast is defined as $\frac{{I}_{ref}-I(x)}{{I}_{ref}+I(x)}$, where *I*(*x*) and *I*_*ref*_ are normalized output signal at the desired point and reference point ($x=+\,resolution$), respectively. According to Fig. 5(b), image information extracted with NFDIC method has larger range of variations around step ($x=0$ nm) than the other methods due to high sensitivity of the phase information to topographical variations, and also dual color schemes provide larger range of variations than the conventional SNOM systems due to the higher field enhancement achieved by utilizing plasmonic nanostructure. Comparing the contrast of different methods shown in Fig. 5(c), it can be clearly seen that NFDIC method provides much larger contrast (about three times larger than dual color schemes, and eighteen times larger than conventional SNOM systems) in imaging a sharp step, as expected, due to extracting the phase information. Also we expect improving our system contrast trough merging the phase information resulting from passage of both hot spots from the step (as described in scanning scenario in Fig. 3), compared to the results plotted in Fig. 5 that are based on phase data obtained from passage of only one hot spot. These results clearly show that extracting phase information in NFDIC technique gives rise to a high contrast and high spatial resolution edge detection.

To conclude, DIC method has been implemented in a near field imaging system. The two main challenges for extension of DIC microscopy to near field microscopy have been addressed, and resolved. The proposed method makes it possible to extract interference signal from the induced near field optical force. Steep phase changes due to variations of the sample topography and its optical properties have been demonstrated.

## Method

In order to quantitatively investigate the proposed idea, numerical simulations were conducted using three-dimensional Method of Moments (MoM) analysis technique. In the simulations, Johnson and Christy dielectric function data^23^ was used for modeling the silver. For the CNT structure, we used the surface conductivity values of planar graphene^24^ in which the mobility was assumed to be 10000 $c{m}^{2}/(V\mathrm{}.s)$, corresponding to the relaxation time of 1 ps for the Fermi energy of 1 eV. Excitation in aperture probes, including dual color probe and conventional aperture probe, and in conventional apertureless probe was considered to be the fundamental mode (HE11) of the probe, and a unit amplitude continuous plane wave with the angle of 60 degrees with respect to the probe axis, respectively. In conventional optical detection mechanism, total electric field intensity in far field region at the angle of 60 degrees with respect to the probe axis was extracted from electromagnetic simulations. In photoinduced force detection mechanism, optical force applied to the probe was calculated using Maxwell stress tensor in which total modulated electric and magnetic fields calculated with the full wave electromagnetic simulations were substituted in Maxwell stress tensor and the z-component of force at the difference modulation frequencies was extracted. Phase changes was also calculated by evaluating zero crossing points of the demodulated optical force.
